# Retracted: The Impact of Graft Nephrectomy on Subsequent Transplants: Multivariate Analysis of Risk Factors for Second Graft Loss and for Multiple Transplantations–A Single-Center Retrospective Study

**DOI:** 10.1155/2016/9782049

**Published:** 2016-10-12

**Authors:** International Scholarly Research Notices

At the request of the authors and the hospital, the article titled “The Impact of Graft Nephrectomy on Subsequent Transplants: Multivariate Analysis of Risk Factors for Second Graft Loss and for Multiple Transplantations–A Single-Center Retrospective Study” [1] has been retracted. The data reported in the article were not reviewed by the Renal Transplant Unit of the Royal London Hospital before submission. The data is not a complete capture and as a result is not an accurate reflection of the effect of the intervention.
